# The incidence of acute kidney injury in very-low-birth-weight infants treated early with caffeine

**DOI:** 10.1007/s00467-025-06694-5

**Published:** 2025-02-03

**Authors:** Shimrit Tzvi-Behr, Noam Schlesinger, Efrat Ben-Shalom, Yaacov Frishberg, Yair Kasirer

**Affiliations:** 1 Division of Pediatric Nephrology, Shaare Zedek Medical Center, Jerusalem, Israel; 2 Faculty of Medicine, Hebrew University of Jerusalem, Jerusalem, Israel; 3 Hadassah Medical Center, Jerusalem, Israel; 4 Neonatal Intensive Care Unit, Shaare Zedek Medical Center, Jerusalem, Israel

**Keywords:** Children, AKI, Prematurity, Caffeine

## Abstract

**Background:**

Acute kidney injury (AKI) in neonates is associated with increased morbidity and mortality, longer hospitalization, and a higher risk for future kidney damage. Caffeine treatment has reportedly been associated with a decreased AKI occurrence. However, previous studies lack uniformity regarding dosage and timing of administration. This study aimed to assess AKI incidence in very-low-birth-weight (VLBW) preterm infants (< 1500 g) treated with early high-dose caffeine and to identify risk factors associated with AKI.

**Methods:**

A retrospective cohort study of VLBW preterm infants admitted to the Neonatal Intensive Care Unit at the Shaare Zedek Medical Center between January 1, 2017, and December 31, 2019. All VLBW infants born < 32 weeks of gestation were treated with a standardized caffeine regimen (20 mg/kg bolus; in the first hour of life, maintenance 10 mg/kg/day). Maternal and infant data including clinical, demographic, and laboratory measurements were retrieved from electronic medical records.

**Results:**

Of 311 VLBW infants admitted, all had adequate serum creatinine and urine output data. Of 301 patients included for analysis, 41 (14%) were diagnosed with AKI, while only 12/301 (4%) were diagnosed during the first week of life. Sixteen infants (5%) had > 1 AKI episode. Seven (7/41, 17%) had AKI stage 1 and seventeen infants (17/41, 42%) had stages 2 and 3. In univariate analysis, sepsis, patent ductus arteriosus, necrotizing enterocolitis (NEC), and hemodynamic instability during the first week of life were more prevalent in the AKI group. Infants with AKI were born with lower birth weights, at earlier gestational weeks, and had lower APGAR and higher CRIB II scores. NEC was the only significant risk factor associated with AKI in multivariate analysis. They also had a higher risk for bronchopulmonary dysplasia (BPD), longer hospitalization, and higher mortality rate.

**Conclusions:**

The incidence of AKI in a cohort of VLBW infants universally treated early with caffeine was 14%, while only 4% had AKI during the first week. Infants with AKI had worse outcomes (BPD and mortality) and longer hospitalization.

**Graphical abstract:**

**Figure:**
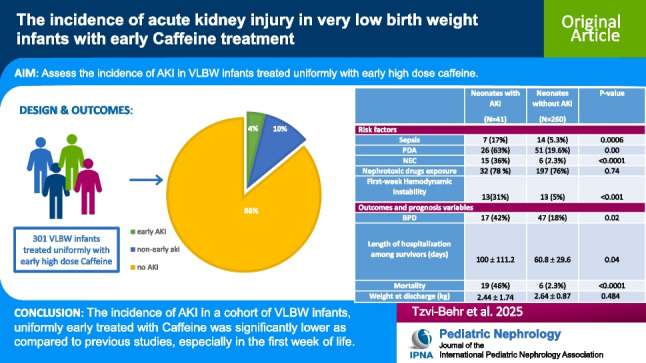
A higher resolution version of the Graphical abstract is available as Supplementary information

**Supplementary Information:**

The online version contains supplementary material available at 10.1007/s00467-025-06694-5.

## Introduction

Nephrogenesis commences on the 5th of gestation and is completed by 32–36 weeks of gestation [1, 2]. As such, when a baby is born prematurely, nephrogenesis will be incomplete and inefficient [3]. Hence, during the first few weeks of life, the kidneys are vulnerable to the effects of various noxious factors such as exposure to nephrotoxic medications, hypoxia, hemodynamic instability, and infections [4]. Moreover, a reduced nephron number can result in decreased renal reserve later in life. Indeed, previous studies showed an association between prematurity and chronic kidney disease [5].

Acute kidney injury (AKI) in preterm infants is associated with prolonged hospitalization, higher morbidity and mortality rates, and an elevated risk for chronic kidney disease [6]. AKI is common among preterm infants, and early AKI (occurring during the first week of life) has been reported between 18 and 40% of cases in previous studies [7–9].

Caffeine citrate, a methylxanthine initially used to treat apnea of prematurity, has been shown to have additional beneficial effects and is widely used to treat premature neonates. Two large studies found a clear association between caffeine treatment and a reduction in early AKI incidence (18% vs. 44% and 11.2% vs. 32%) [6, 10]. In the present study, we aim to describe the current incidence of AKI episodes and assess AKI risk factors in a cohort of very-low-birth-weight (VLBW) neonates treated early with caffeine.

## Methods

All neonates that were hospitalized in the Neonatal Intensive Care Unit (NICU) of the Shaare Zedek Medical Center from January 1, 2017, to December 31, 2019, with a birth weight of less than 1500 g (VLBW) were eligible for inclusion in this retrospective cohort study. Ethical approval for the study was provided by the Institutional Review Board, which waived the need for informed consent based on the strict maintenance of participants’ anonymity.

Exclusion criteria included newborns with inadequate data (defined as fewer than two serum creatinine measurements or fewer than two full urine reports), newborns hospitalized for less than 48 h or admitted after 1 week of postnatal age, and/or newborns with major congenital or chromosomal anomalies. Maternal and infant clinical, demographic, and laboratory data for the entire hospitalization were retrieved from our electronic medical records. Serum creatinine is routinely measured in any specimen sent for biochemistry laboratory analysis, at least every 2 days while on total parenteral nutrition and routinely in the first 2 weeks of life. Thereafter creatinine is measured at least twice monthly. Urine output is routinely reported quantitatively in the intensive care unit and qualitatively after transfer to the step-down unit, which occurs when the infant is stable and at least 33 weeks’ corrected age. We excluded the daily urine report of the first day of life from the analysis.

Caffeine is routinely administered intravenously in the first hour of life at a loading dose of 20 mg per kg per day, followed by a maintenance dose of 10 mg per kg per day, to all preterm infants born at less than 32 weeks of gestation, until 35 weeks post-conceptual age (PCA) or 5 days apnea free.

The Clinical Risk Index for Babies (CRIB II) scoring system is used to assess neonatal illness severity and has been shown to be predictive of mortality in preterm infants. It is composed of five variables: birth weight, sex, body temperature, base excess, and gestational age [11].

AKI was defined and severity classified according to the neonatal kidney disease improving global outcomes (KDIGO) criteria (Table 1) [12]. Early AKI was defined as AKI occurring during the first week of life.

**Table 1:** Neonatal AKI KDIGO classification

Stage	Serum creatinine	Urine output
0	No change or rise $<$ 0.3 mg/dL	$\ge$ 0.5 mL/kg/h
1	SCr rise $\ge$ 0.3 mg/dL within 48 h or rise $\ge$ 1.5–1.9 $\times$ reference SCr* within 7 d	$<$ 0.5 mL/kg/h for 6 to 12 h
2	SCr rise $\ge$ 2–2.9 $\times$ reference SCr**	$<$ 0.5 mL/kg/h for $\ge$ 12 h
3	SCr rise $\ge$ 3 $\times$ reference SCr** or SCr $\ge$ 2.5 mg/dL or receipt of dialysis	$<$ 0.3 mL/kg/h for $\ge$ 24 h or anuria for $\ge$ 12 h

*Scr*, serum creatinine; *the lowest SCr value in the last 7 days; **the lowest previous SCr value

AKI risk factors prior to AKI diagnosis included culture-proven sepsis (diagnosed when a pathogen was isolated from blood or cerebrospinal fluid), patent ductus arteriosus (PDA) (hemodynamically significant and treated medically), necrotizing enterocolitis (NEC) (determined by the clinical and radiological criteria of Bell et al. [13], and only definite NEC (Bell stages II–III)), nephrotoxic drug exposure (gentamycin, amikacin, vancomycin, and ibuprofen), and hemodynamic instability (defined as a need for cardiovascular support, either inotropic agents or saline bolus) were evaluated.

Long-term prognostic factors including the length of hospitalization, bronchopulmonary dysplasia (BPD) (diagnosed according to the criteria of Bancalari et al., including clinical and radiological features, together with the requirement for oxygen therapy at 36 weeks postmenstrual age) [14], weight at discharge, and mortality were further evaluated in association with AKI occurrence.

When coagulase-negative staphylococcal species (CoNS) were isolated from blood, definite sepsis was diagnosed if either two time-separated cultures of the same species and the infant had been treated with antibiotics for ≥ 5 days, or when a single CoNS species was isolated in association with clinical signs and treatment with ≥ 5 days of antibiotics.

Statistical analysis was performed using the IBM SPSS Statistics 26.0 software package (IBM SPSS Statistics for Windows, Version 26.0. Armonk, NY). To compare distributions of perinatal factors and neonatal morbidities between infants with and without AKI, the chi-square test was used for categorical variables and the *t*-test for continuous variables.

## Results

During the study period, 311 eligible newborns were admitted to the NICU. Of these, 8 were excluded from the study due to birth defects, and 2 neonates were excluded because they were admitted later than 7 days of life. There were no exclusions for missing data. A total of 301 neonates were included in the final analysis. All infants received the prescribed caffeine dose.

Patients were divided into two groups: those diagnosed with AKI and those without. Patients’ characteristics are presented in Table 2. Patients diagnosed with AKI were born earlier, with lower birth weight and Apgar scores, and had higher CRIB II scores. Forty-one neonates (14%) had at least one event of AKI during hospitalization. The median age at the first AKI episode was 11 days (IQR 6–20).

**Table 2:** Patients’ characteristics (all continuous presented as mean (± standard deviation) and all categorical data are presented as number (percentage))

Patients' characteristics	Neonates with AKI (*N* = 41)	Neonates without AKI (*N* = 260)	*P* value
Birth weight (gm)	827.6 ± 211.4	11,973. ± 226.4	< 0.01
Gestational age (weeks)	260. ± 0.4	29.8 ± 2.5	< 0.01
Sex			
Female	14 (34%)	134(51%)	0.04
Apgar score			
1 min > 7	14 (34%)	164 (64%)	0.001
5 min > 7	27 (66%)	228 (88%)	0.001
CRIB II score	11.6 ± 3.1	6.4 ± 3.1	< 0.01
Maternal risk factors			
Eclampsia	4 (10%)	17 (6%)	0.5
Chorioamnionitis	3 (7%)	25 (10%)	0.78
PPROM	12 (29%)	55 (21%)	0.24
Placenta abruption/previa	15 (36%)	55 (21%)	0.031

*AKI*, acute kidney injury; *PPROM*, preterm prelabor rupture of membranes

**Fig. 1:**
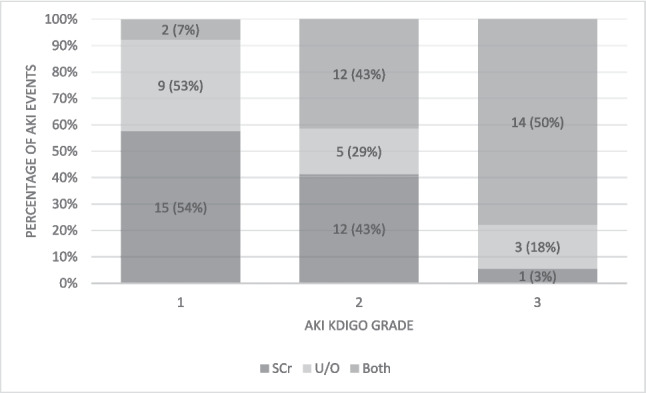
KDIGO AKI grade and diagnostic criteria met. AKI, acute kidney injury; Scr, serum creatinine; U/O, urinary output

Of these, 30 (30/41, 73%) neonates met both creatinine and urine output criteria, 9 (22%) had non-oliguric AKI, and 2 (5%) infants were diagnosed with AKI based solely on urine output as depicted in Fig. 1. Sixteen newborns (39%) had two or more AKI events. Seven neonates (17%) had AKI stage 1, and the remaining 34 were equally divided between stages 2 and 3 (17, 41%). Only 4% (12/301) of enrolled infants developed AKI in the first week of life.

There were 73 AKI episodes during the study period (stage 1, 26 (35%); stage 2, 29 (40%); and stage 3, 18 (25%)).

## Risk factors and prognostic factors associated with acute kidney injury

Risk factors associated with AKI are presented in Table 3. In univariate analysis sepsis, PDA, NEC, and first-week hemodynamic instability were more prevalent in the AKI group. Infants with AKI had a longer hospitalization and higher BPD and mortality rate.

**Table 3:** Univariate analysis of risk factors, outcome and prognosis indicators associated with AKI (all continuous presented as mean (± standard deviation) and all categorical data are presented as number (percentage))

	Neonates with AKI (*N* = 41)	Neonates without AKI (*N* = 260)	*P*-value
Risk factors			
Sepsis	7 (17%)	14 (5.3%)	0.0006
PDA	26 (63%)	51 (19.6%)	0.00
NEC	15 (36%)	6 (2.3%)	< 0.0001
Nephrotoxic drugs exposure	32 (78%)	197 (76%)	0.74
First-week hemodynamic instability	13(31%)	13 (5%)	< 0.001
Outcomes and prognosis variables			
BPD	17 (42%)	47 (18%)	0.02
Length of hospitalization among survivors (days)	100 ± 111.2	60.8 ± 29.6	0.04
Mortality	19 (46%)	6 (2.3%)	< 0.0001
Weight at discharge (kg)	2.44 ± 1.74	2.64 ± 0.87	0.484

*AKI*, acute kidney injury; *CRIB II*, Clinical Risk Index for Babies scoring system; *PDA*, patent ductus arteriosus; *BPD*, bronchopulmonary dysplasia; *NEC*, necrotizing enterocolitis

In multivariate analysis adjusted for sex, since the female sex is known to be a protective factor for neonatal comorbidities, we examined the effect of each risk factor on AKI occurrence (Supplementary Table 1). NEC was the only significant risk factor associated with AKI.

In a second multivariate analysis for predicting mortality, only AKI remained a significant risk factor for mortality (OR 68.6; 95% CI 6.45–729.6; *p*-value < 0.001) (Supplementary Table 2).

**Fig. 2:**
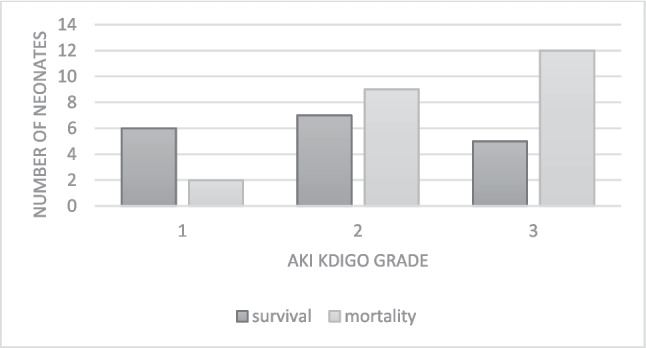
Mortality and survival rate according to KDIGO AKI grade classification. AKI, acute kidney injury

Mortality rates and survival by AKI stages are depicted in Fig. 2. Out of 41 neonates diagnosed with AKI, only 17 (41%) survived. Among AKI survivors, hospitalization length was 183 ± 96 days (median, IQR), last creatinine and blood pressure (BP) measurements were 0.23 ± 0.06 mg/dl, and mean systolic and diastolic BP were 87 ± 17 and 49 ± 12 mmHg, respectively.

## Discussion

Our study shows low AKI rates in VLBW neonates admitted to our tertiary medical center. Compared to previous reports, the rate of AKI incidence during the entire hospitalization period was 14%, significantly lower than 18–40% reported in previous studies [7–9] (*p* < 0.001, *p* < 0.048 respectively). Furthermore, early onset AKI was lower in our study (4% vs. 14–26% [9], *p* < 0.001). Furthermore, in our data, 29% of all AKI cases occurred in the first week of life as compared to about two-thirds in previous reports (*p* < 0.001). AKI was found to be associated with higher mortality (59% vs. 2%, *p* < 0.0001). Both creatinine and urine output criteria for the diagnosis of AKI were met in most newborns (73%), and in 5%, the AKI urine output criterion was solely met. Given the multitude of creatinine measurements, full urine output reports, and adequate data on all enrolled neonates, under-diagnosis of AKI in our cohort is unlikely.

Similar to other studies, gestational age, birth weight, Apgar score, CRIB II score, and sepsis were found to be associated with AKI in a univariate analysis. However, only NEC remained significant in the multivariate analysis, suggesting that AKI is mainly secondary to other fulminant events that compromise the preterm infant.

Lower AKI rates found in this study are in line with a recently published study by Chen et al. [15], reporting a declining incidence of AKI across a 14-year period possibly explained by improved neonatal care, which we speculate reflects in part the widespread use of caffeine.

Caffeine’s nephro-protective effect is plausible given its effect on the kidneys including increased renal blood flow and tissue oxygenation, enhanced sodium excretion, and elevated glomerular filtration rate [16]. In a recent review published by Yang et al., different mechanisms by which caffeine can reduce AKI occurrence, including reducing oxidative stress, contributing to mitochondrial homeostasis, and inhibiting endoplasmic reticulum stress, were suggested [17]. Methylxanthines, such as caffeine and aminophylline, were correlated with decreased AKI incidence in asphyxiated term infants [18], preterm cohort, VLBW infants, and specifically in preterms diagnosed with NEC [19]. Carmody et al. reported a reduced risk of AKI in VLBW infants treated with caffeine [10]. However, a limitation of this study was the lack of identification of potential AKI causes. In a reanalysis of the Assessment of Worldwide Acute Kidney Injury Epidemiology in Neonates study by Harer et al., an early AKI incidence of 11.2% in caffeine-treated preterm infants was reported, as compared to 32% in caffeine-naive infants [6]. However, the timing of caffeine in their study was not uniform, and doses were not presented. Herein lies the importance of the present work which described the incidence of AKI in a cohort of VLBW infants uniformly treated with early high-dose caffeine. In a recent study by Harer et al., caffeine use in extremely premature infants stratified for having BPD or not, for each additional week of caffeine, the no-BPD group had a 21% decreased adjusted odds of eGFR < 90 ml/min/1.73 m^2^ (OR 0.78; CI 0.62–0.99) at 2 years of age. Hence, caffeine use in extremely low gestational age infants and improved kidney function is further supported [20].

This study is limited by its retrospective design. The lack of proper controls that were not exposed to caffeine allows us to only speculate about the protective effect of caffeine against AKI in preterm infants. Clearly, the lower incidence of AKI can be a result of multiple factors, and any comparison to previous cohorts is biased by period effects. Also, our patients could be less sick than populations enrolled in other studies. However, caffeine’s role is supported by other studies [10, 11, 20].

Our main goal was to better describe the current rate of AKI among premature infants treated with an early standard dose of caffeine. Preterm infants are a vulnerable population with an increased risk for future kidney disease. Monitoring AKI incidence is critical for identifying appropriate measurements to prevent kidney insults. Further studies are needed to support the protective effect of early caffeine treatment.

## Supplementary Information

Below is the link to the electronic supplementary material.

Graphical abstract (PPTX 94 KB)

Supplementary file2 (DOCX 19 KB)

Supplementary file3 (XLSX 55 KB)

## Data Availability

The data analyzed during the current study are included in the supplemental table. Additional data are available from the corresponding author upon reasonable request.

## Abbreviations

AKI: Acute kidney injury
VLBW: Very low birth weight
NICU: Neonatal Intensive Care Unit
KDIGO: Kidney Disease Improving Global Outcomes
GFR: Glomerular filtration rate
RDS: Respiratory distress syndrome
PCA: Post conceptual age
CRIB: Clinical Risk Index for Babies scoring system
Scr: Serum creatinine
GA: Gestational age
SGA: Small for gestational age
AGA: Appropriate for gestational age
LGA: Large for gestational age
PPROM: Preterm prolonged rupture of membranes
NEC: Necrotizing enterocolitis
IVH: Intraventricular hemorrhage
BPD: Bronchopulmonary dysplasia
PDA: Patent ductus arteriosus
UOP: Urine output
