# Supplementary material for: The incidence of acute kidney injury in very-low-birth-weight infants treated early with caffeine
Source: Pediatr Nephrol. 2025 Feb 3;40(6):2091–6. doi: 10.1007/s00467-025-06694-5 (PMC12031835; doi:10.1007/s00467-025-06694-5)
Supplement: Supplementary file 1 — Graphical abstract (PPTX 94 KB) [file 467_2025_6694_MOESM1_ESM.pptx]

## Slide 1
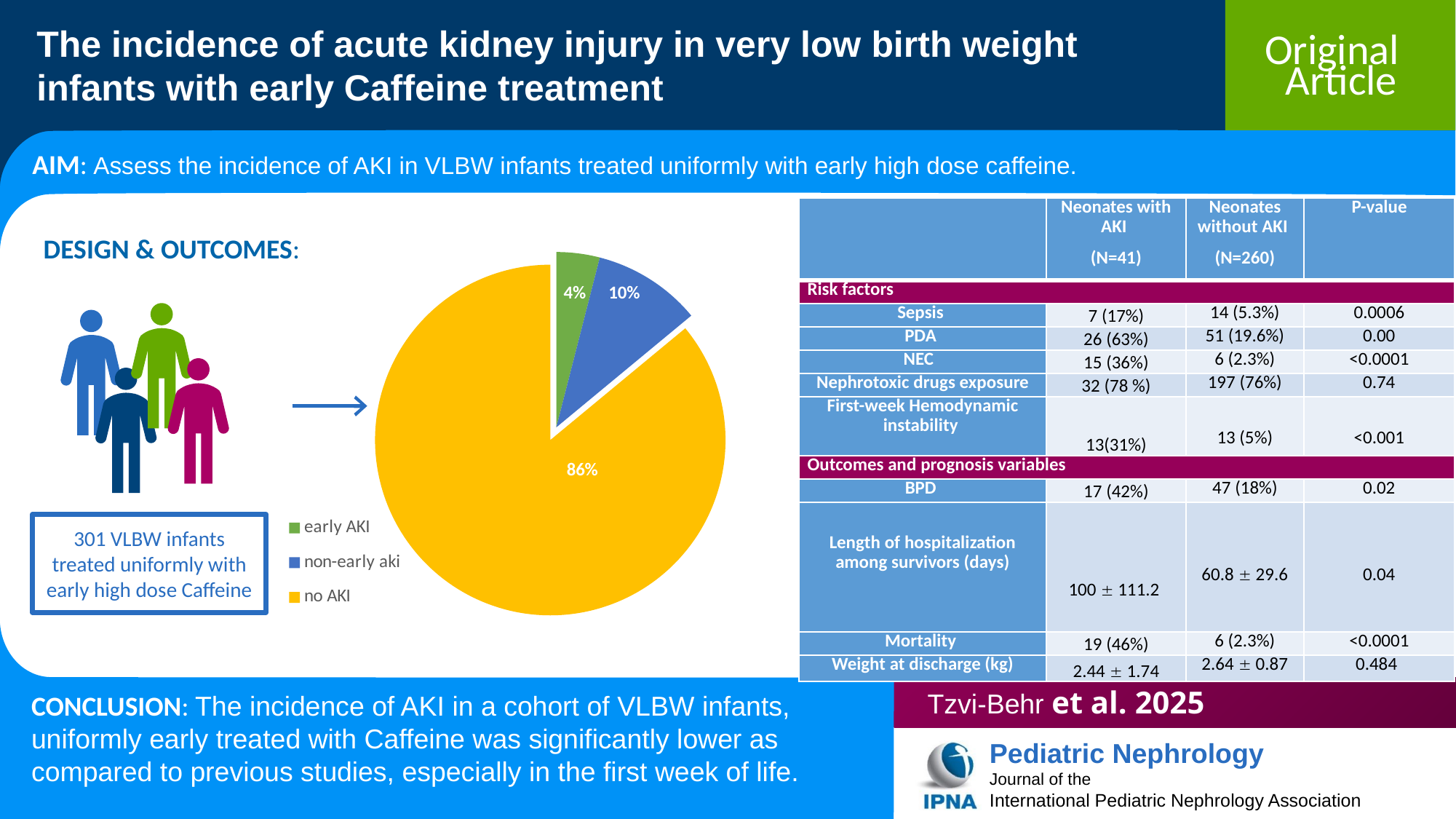

The incidence of acute kidney injury in very low birth weight infants with early Caffeine treatment
AIM: Assess the incidence of AKI in VLBW infants treated uniformly with early high dose caffeine.
### Chart
| Category | Sales |
|---|---|
| early AKI | 0.04 |
| non-early aki | 0.1 |
| no AKI | 0.86 || | Neonates with AKI (N=41) | Neonates without AKI (N=260) | P-value |
| --- | --- | --- | --- |
| Risk factors | | | |
| Sepsis | 7 (17%) | 14 (5.3%) | 0.0006 |
| PDA | 26 (63%) | 51 (19.6%) | 0.00 |
| NEC | 15 (36%) | 6 (2.3%) | <0.0001 |
| Nephrotoxic drugs exposure | 32 (78 %) | 197 (76%) | 0.74 |
| First-week Hemodynamic instability | 13(31%) | 13 (5%) | <0.001 |
| Outcomes and prognosis variables | | | |
| BPD | 17 (42%) | 47 (18%) | 0.02 |
| Length of hospitalization among survivors (days) | 100 ± 111.2 | 60.8 ± 29.6 | 0.04 |
| Mortality | 19 (46%) | 6 (2.3%) | <0.0001 |
| Weight at discharge (kg) | 2.44 ± 1.74 | 2.64 ± 0.87 | 0.484 |
DESIGN & OUTCOMES:
301 VLBW infants treated uniformly with early high dose Caffeine
Tzvi-Behr et al. 2025
CONCLUSION: The incidence of AKI in a cohort of VLBW infants, uniformly early treated with Caffeine was significantly lower as compared to previous studies, especially in the first week of life.
-----
