# Supplementary material for: The incidence of acute kidney injury in very-low-birth-weight infants treated early with caffeine
Source: Pediatr Nephrol. 2025 Feb 3;40(6):2091–6. doi: 10.1007/s00467-025-06694-5 (PMC12031835; doi:10.1007/s00467-025-06694-5)
Supplement: Supplementary file 2 — Supplementary file2 (DOCX 19 KB) [file 467_2025_6694_MOESM2_ESM.docx]

|  | Odds ratio | 95% CI for OR | P-value |
| --- | --- | --- | --- |
| Sex | 0.11 | 0.02–0.51 | 0.005 |
| Gestational age | 0.61 | 0.31–1.21 | 0.16 |
| SGA/AGA/LGA | - | - | 0.92 |
| APGAR Score (< 7 at 1 min) | 0.78 | 0.17–3.59 | 0.75 |
| APGAR Score (< 7 at 5 min) | 1.02 | 0.17–3.59 | 0.98 |
| CRIB Score | 1.36 | 0.85–2.17 | 0.2 |
| NEC | 18.8 | 2.78–127 | 0.03 |
| PDA | 2.24 | 0.58–8.64 | 0.24 |
| First-week hemodynamic instability | 1.7 | 0.31–9.19 | 0.54 |
| Sepsis | 0.47 | 0.08–2.98 | 0.43 |

**Supplementary Table 1** : Multivariate logistic regression model for predicting AKI

Abbreviations: AKI- acute kidney injury, OR- odds ratio, CI- confidence interval, SGA- small for gestational age, AGA- appropriate for gestational age, LGA- large for gestational age, CRIB- Clinical Risk Index for Babies scoring system, NEC- necrotizing enterocolitis, PDA- patent ductus arteriosus

|  | Odds ratio | 95% CI for OR | P-value |
| --- | --- | --- | --- |
| Sex | 2.19 | 0.4–12.14 | 0.37 |
| Gestational age | 1.43 | 0.61–3.37 | 0.41 |
| SGA/AGA/LGA | - | - | 0.46 |
| APGAR Score (< 7 at 1 min) | 0.4 | 0.06–2.86 | 0.36 |
| APGAR Score (< 7 at 5 min) | 2.35 | 0.37–14.86 | 0.36 |
| CRIB Score | 1.44 | 0.79–2.62 | 0.23 |
| NEC | 1.8 | 0.26–12.65 | 0.55 |
| PDA | 1.07 | 0.17–6.86 | 0.95 |
| AKI | 68.6 | 6.45–729.6 | <0.001 |
| First-week hemodynamic instability | 2.51 | 0.36–17.49 | 0.35 |

**Supplementary Table 2**: Multivariate logistic regression model for predicting mortality

Abbreviations: OR- odds ratio, CI- confidence interval, SGA- small for gestational age, AGA- appropriate for gestational age, LGA- large for gestational age, CRIB- Clinical Risk Index for Babies scoring system, NEC- necrotizing enterocolitis, PDA- patent ductus arteriosus, AKI- acute kidney injury
